# Astragaloside IV modulates TGF‐β1‐dependent epithelial‐mesenchymal transition in bleomycin‐induced pulmonary fibrosis

**DOI:** 10.1111/jcmm.13725

**Published:** 2018-07-04

**Authors:** Weibin Qian, Xinrui Cai, Qiuhai Qian, Wei Zhang, Dongli Wang

**Affiliations:** ^1^ Affiliated Hospital of Shandong University of Traditional Chinese Medicine Jinan Shandong China; ^2^ Shandong Academy of Occupational Health and Occupational Medicine Shandong Academy of Medical Sciences Jinan Shandong China

**Keywords:** astragaloside IV, epithelial‐mesenchymal transition, forkhead box O3a, idiopathic pulmonary fibrosis, transforming growth factor‐β1

## Abstract

Epithelial‐mesenchymal transition (EMT) plays an important role in idiopathic pulmonary fibrosis (IPF). Astragaloside IV (ASV), a natural saponin from astragalus membranaceus, has shown anti‐fibrotic property in bleomycin (BLM)‐induced pulmonary fibrosis. The current study was undertaken to determine whether EMT was involved in the beneficial of ASV against BLM‐induced pulmonary fibrosis and to elucidate its potential mechanism. As expected, in BLM‐induced IPF, ASV exerted protective effects on pulmonary fibrosis and ASV significantly reversed BLM‐induced EMT. Intriguing, transforming growth factor‐β1 (TGF‐β1) was found to be up‐regulated, whereas Forkhead box O3a (FOXO3a) was hyperphosphorylated and less expressed. However, ASV treatment inhibited increased TGF‐β1 and activated FOXO3a in lung tissues. TGF‐β1 was administered to alveolar epithelial cells A549 to induce EMT in vitro. Meanwhile, stimulation with TGF‐β1‐activated phosphatidylinositol 3 kinase/protein kinase B (PI3K/Akt) pathway and induced FOXO3a hyperphosphorylated and down‐regulated. It was found that overexpression of FOXO3a leading to the suppression of TGF‐β1‐induced EMT. Moreover, ASV treatment, similar with the TGF‐β1 or PI3K/Akt inhibitor, reverted these cellular changes and inhibited EMT in A549 cells. Collectively, the results suggested that ASV significantly inhibited TGF‐β1/PI3K/Akt‐induced FOXO3a hyperphosphorylation and down‐regulation to reverse EMT during the progression of fibrosis.

## 1 INTRODUCTION

Idiopathic pulmonary fibrosis (IPF) is a prototype of chronic, progressive and fibrotic lung disease.^1^ Incidence of IPF has risen over time. Although disease course is variable and somewhat unpredictable, the median survival time from diagnosis is 2‐4 years.^2^ IPF was typically characterized by excessive production and deposition of extracellular matrix (ECM), chronic inflammatory process, remodelling of abnormal lung tissue structure and the gradual decline of pulmonary function.^3^ The current notion Myofibroblasts derived from epithelial cells by epithelial–mesenchymal transition (EMT) exhibit abnormal proliferation and ECM overproduction, resulting in the development of IPF.^4^ And studies found approximately one‐third of fibroblasts are of epithelial origin in pulmonary fibrosis.^5^ When EMT was occurring in the lung, the E‐cadherin in epithelial cells decreased and α‐smooth muscle actin (α‐SMA) mesenchymal cells increased.^6^ EMT plays a critical role in the development of pulmonary fibrosis. The cytokine transforming growth factor‐β (TGF‐β) functions served as an important mediator of fibrogenesis. TGF‐β1 induces the activation of fibroblasts to undergo a phenotypic transition to myofibroblasts, which are the effectors of the fibrotic state.^7^ A plenty of works have identified that TGF‐β1 is an important pro‐fibrotic factor that has been shown to induce EMT in pulmonary fibrosis.^8^ TGF‐β1‐induced EMT is mainly mediated by Smad‐dependent or Smad‐independent pathways.^9^ Thus, anti‐EMT pathway or the method of inhibiting of TGF‐β1 signalling could provide a novel potential target for the treatment of IPF.

Astragaloside IV (ASV) is one of the major and main active substances of traditional Chinese medicinal plant astragalus membranaceus. Recently, studies on the pharmacological effect of ASV have demonstrated its antioxidant, anti‐inflammatory, anti‐diabetes, anti‐hypertensive, anti‐asthma, and anti‐fibrotic properties.^10^ ASV was found to alleviate the progression of kidney fibrosis via inhibition of MAPK pathway and TGF‐β/Smad signalling pathway.^11^, ^12^ Besides, ASV could protect against TGF‐β1‐induced EMT by inhibiting in peritoneal mesothelial cells by promoting smad 7 expression.^13^ These all indicated the great potential of the anti‐fibrosis and anti‐EMT activity of ASV. Bleomycin (BLM), a drug widely used as an antineoplastic, causes a dose‐dependent interstitial pulmonary fibrosis.^14^ BLM‐induced pulmonary fibrosis is the most commonly used animal model of IPF for studying disease pathogenesis and testing of novel pharmaceutical compounds.^15^ Researchers have identified that ASV could attenuate BLM‐induced pulmonary fibrosis by suppressing oxidative stress and inflammation.^16^ In addition, a recent study found that ASV significantly inhibited BLM‐induced ECM deposition.^17^ However, whether the anti‐pulmonary fibrosis of ASV was associated with its inhibition of EMT during pulmonary fibrosis is still unknown.

Forkhead box O (FOXO) transcription factors are involved in multiple physiological and pathological processes, including apoptosis, ageing, proliferation, metabolism, immunity and differentiation.^18^ The mammalian FOXO have 4 members: FOXO1, FOXO3, FOXO4 and FOXO6. FOXO3a are expressed in most tissues and cells, including fibroblasts.^19^ Accumulation of collagen and increased expression of α‐SMA is associated with FOXO3a inactivation.^20^ Moreover, suppressed FOXO3a activity resulting from hyperphosphorylation of FOXO3a by Akt was found closely linked to the progression of IPF.^21^ However, limited evidence linked the relationship between FOXO3a and EMT in pulmonary fibrosis.

Considering the anti‐fibrotic effect of ASV in BLM‐induced pulmonary fibrosis, the possible mechanisms of that effect and the characteristics of EMT in pulmonary fibrosis, we hypothesized that ASV could suppress TGF‐β1‐induced phosphorylation of Akt and inactivity of FOXO3a and subsequently inhibit EMT. Here, we used BLM to reproduce IPF in rats and induced EMT alveolar epithelial cells via TGF‐β1 treatment to investigate the potential effects of ASV on EMT and its underlying mechanisms.

## 2 MATERIALS AND METHODS

### 2.1 Ethics statement

All of the animal procedures including housing, care and experimental protocols were approved by the Animal Care and Use Committee of Shandong University of Traditional Chinese Medicine. All procedures on the mice were performed in accordance with the guidelines from the National Institutes of Health.

### 2.2 Pulmonary fibrosis model and treatment

Forty rats were randomly divided into 4 groups. Control group: the rats were given a single intratracheal instillation of 50 mL saline; BLM group: the rats were given a single intratracheal instillation of 50 mL saline containing BLM (5 mg/kg, Nippon Kayaku, Japan); ASV group: the saline‐instilled rats treated with ASV (20 mg/kg; Sigma‐Aldrich, St. Louis, MO, USA) starting at day 15 and treated daily for 14 days by intragastric administration; ASV + BLM group: the BLM‐instilled rats treated with ASV starting 15th day after BLM treatment through administration by gavage for 14 days; all rats were killed by intraperitoneal injection of 10% chloral hydrate (5 mL/kg body weight) at the 28^th^ day after treatment. After 28 days, all rats were killed, and the lung tissues were fixed or frozen for subsequent pathology testing.

### 2.3 Cell culture and treatment

The human type II alveolar epithelial cells (A549) were purchased from American Type Culture Collection (ATCC, Rockville, Maryland, USA). Cells grew in Dulbecco's modified Eagle's medium (DMEM, Hyclone, USA) supplemented with 10% fetal bovine serum (Gibco, MD, USA). Cells were incubated at 37°C with 5% CO_2_. Cells were incubated with recombinant human TGF‐β1 (10 ng/mL, R&D Systems, Minneapolis, MN) for various time (0.25 hour, 0.5 hour, 1 hour, 2 hours, 24 hours or 48 hours). To induce the EMT cell model, A549 cells were maintained in growth media supplemented with 10 ng/mL TGF‐β1 for 48 hours, as previously described.^22^ ASV (Xiya Reagent, Shandong, China) was dissolved in DMSO at the final concentration of 100 μg/mL. Cells were also pre‐treated with TGF‐β1 inhibitor SB431542 (10 μmol/L, MedChemExpress, Monmouth Junction, NJ, USA) for 30 minutes or a specific PI3K/Akt inhibitor LY 294002 (20 μmol/L, MedChemExpress) before treatment with TGF‐β1 in some experiments.

### 2.4 Histologic analyses and Immunohistochemistry

The pulmonary tissues were prepared by being submerged in formaldehyde solution (10%) and embedded in paraffin. The paraffin blocks were cut at 5 μm using microtome. The sections stained with hematoxylin and eosin (H&E) staining and Masson's trichrome staining following the manufacturers’ instructions. Immunohistochemistry was used to investigate the expression of α‐SMA and FOXO3a using GTVisionTM + Detection System/Mo&Rb (Gene Tech, Shanghai, China) according to instruction book. After water‐bath heating for antigen retrieval in 0.01 mol/L citrate buffer (pH 6.0), 30% H2O2 was added to the sections to inactivate the endogenous enzymes at room temperature for 10 minutes. Then, the slides were blocked with 5% BSA blocking solution at room temperature for 15 minutes and incubated with primary anti‐α‐SMA (1:400) and anti‐FOXO3a (1:500) antibodies overnight at 4°C. Negative controls were performed by omitting the primary antibody. Subsequently, the sections were incubated with general horseradish peroxidase‐labelled secondary antibody at working fluid for 30 minutes at 37°C, and developed with diaminobenzidine (DAB) for colour reaction. The nuclear staining with hematoxylin and then using a light microscope (Leica DM3000, Leica Company, Germany).

### 2.5 Immunofluorescence

The previous procedures of immunofluorescence for lung tissues were the same as immunohistochemistry. After incubated with anti‐E‐cadherin or α‐SMA (1:500) antibody at 4°C overnight, the sections were incubated with Alexa 488 Goat Anti‐rabbit IgG and Alexa 594 Goat Anti‐Rat IgG at room temperature for 1 hour. Nuclei were counterstained with DAPI, and the sections were then captured under a fluorescence microscope. For in vitro immunofluorescence, the cells were fixed with 4% paraformaldehyde for 15 minutes, and permeabilization with 0.1% Triton‐X 100. After blocking with 5% BSA for 30 minutes at room temperature, the cells were incubated with the anti‐E‐cadherin antibody (1:500), α‐SMA antibody (1:400) and FOXO3a (1:400) at 4°C overnight. After rinsed with PBS, cells were incubated with Alexa 594 Goat Anti‐Rat IgG2A or Alexa 488 Goat Anti‐rabbit IgG. All secondary antibodies were purchased from Invitrogen (Carlsbad, CA, USA) and were used at a working dilution of 1:500. 6‐diamidino‐2‐phenylindole (DAPI) (Life Technologies Corporation) was used to identify the nucleus. The images were captured by an Nikon Eclipse 800 epifluorescence microscope with the appropriate filters.

### 2.6 Measurement of oxidative stress and inflammation in lung tissues

The right lung of the rats was homogenized in 50 mmol/L PBS containing 0.5% hexadecylammonium bromide and 5 mmol/L EDTA. After the lung extracts were centrifuged at 12 000 *g* for 15 minutes at 4°C, the supernatants were acquired. For measurement of oxidative stress, the appropriate volume of supernatant was collected to determine the release of MDA, SOD and GSH‐Px using Malondialdehyde (MDA) assay kit, Superoxide Dismutase (SOD) assay kit and Glutathione Peroxidase Assay Kit (Jian Cheng Biological Engineering Institute, Nanjing, China) according to the manufacturer's instructions. To determine the inflammatory response, the levels of TNF‐α and IL‐6 were measured using the corresponding ELISA kit (R&D Systems, Minneapolis, MN, USA) following the manufacturer's instructions.

### 2.7 RNA extraction and quantitative real‐time PCR (qRT‐PCR)

Total RNA was isolated using TRIzol Reagent (Takara, Shiga, Japan). The PrimeScriptTM RT‐PCR Kit (Takara) was used to process first‐strand cDNA synthesis. The mRNA of COLIA1, TGF‐β1 and FOXO3a were evaluated using the SYBR Green PCR Master Mix (Takara) on an iCyler iQ Real‐Time PCR System (Bio‐Rad Laboratories Inc., USA) according to the manufacturer's instructions with the following reaction conditions: 94°C for 2 minutes, followed by 40 cycles at 94°C for 10 seconds, 60°C for 1 minutes, and 30 seconds at 72°C. GAPDH was used as internal controls. The sequences used were listed in Table 1. We calculated the CT values of each gene in samples and used the 2^−ΔΔCt^ method to measure the transcript levels.

**Table 1:** The forward and reverse primers for real‐time PCR

Gene name		Sequence
COLIA1	Forward	TGGTGAGACGTGGAAACCTG
	Reverse	CTTTGCATAGCACGCCATCG
TGF‐β1	Forward	TGATCCAGATGCGCTGTGGCTT
	Reverse	CTCAGTAAAGGAGAGCAATTCT
FOXO3a	Forward	CTCCACCCCTGCTGAGATGAT
	Reverse	AGTGAGAACGTTGTCCCGCGCTGG
GAPDH	Forward	TGCACCACCAACTGCTTAGC
	Reverse	GGCATGGACTGTGGTCATGAG

### 2.8 Western blotting

Total protein was extracted from frozen lung tissue or cells using 1% RIPA Lysis Buffer (Beyotime, Jiangsu, China) supplemented with protease/phosphatase inhibitor cocktail (Sigma‐Aldrich; St. Louis, MO, USA). The BCA method was used to determine protein concentrations and the lysates were mixed with 10% SDS‐PAGE then electrophoresis was performed on the same volume. The protein was then transferred onto polyvinylidene fluoride membranes (Bio‐Rad) and was blocked with 5% skim milk for 1 hour at room temperature. The membranes were incubated with primary antibodies against Col1a (1:1000), α‐SMA (1:1000), E‐cadherin (1:1000), TGF‐β1 (1:500), Phospho‐FOXO3a (Ser253), Phospho‐FOXO3a (Thr32), FOXO3a (1:500) and GAPDH (1:1000, all from Abcam, Cambridge, MA, USA) antibodies overnight at 4°C. Subsequently, the blots were washed with PBST and incubated for 1 hour at room temperature with an anti‐rabbit IgG horseradish peroxidase conjugated antibody (1:5000; Abcam). Signals were developed using Super Signal west pico kit (Bridgen Biological Technology, Shanghai, China) and the intensities of the signals were quantified using ImageJ software 6.0.

### 2.9 Statistical analysis

Experimental results were expressed as mean ± standard deviation. Differences were analysed by one‐way ANOVA using SPASS 20.0 (SPSS, Inc., Chicago, IL, USA) and GraphPad Prism 5.0 (GraphPad Software Inc., CA) was used to generate graphs. *P* < .05 was considered statistically significant.

## 3 RESULTS

### 3.1 ASV alleviated BLM‐induced pulmonary fibrosis

**Figure 1:**
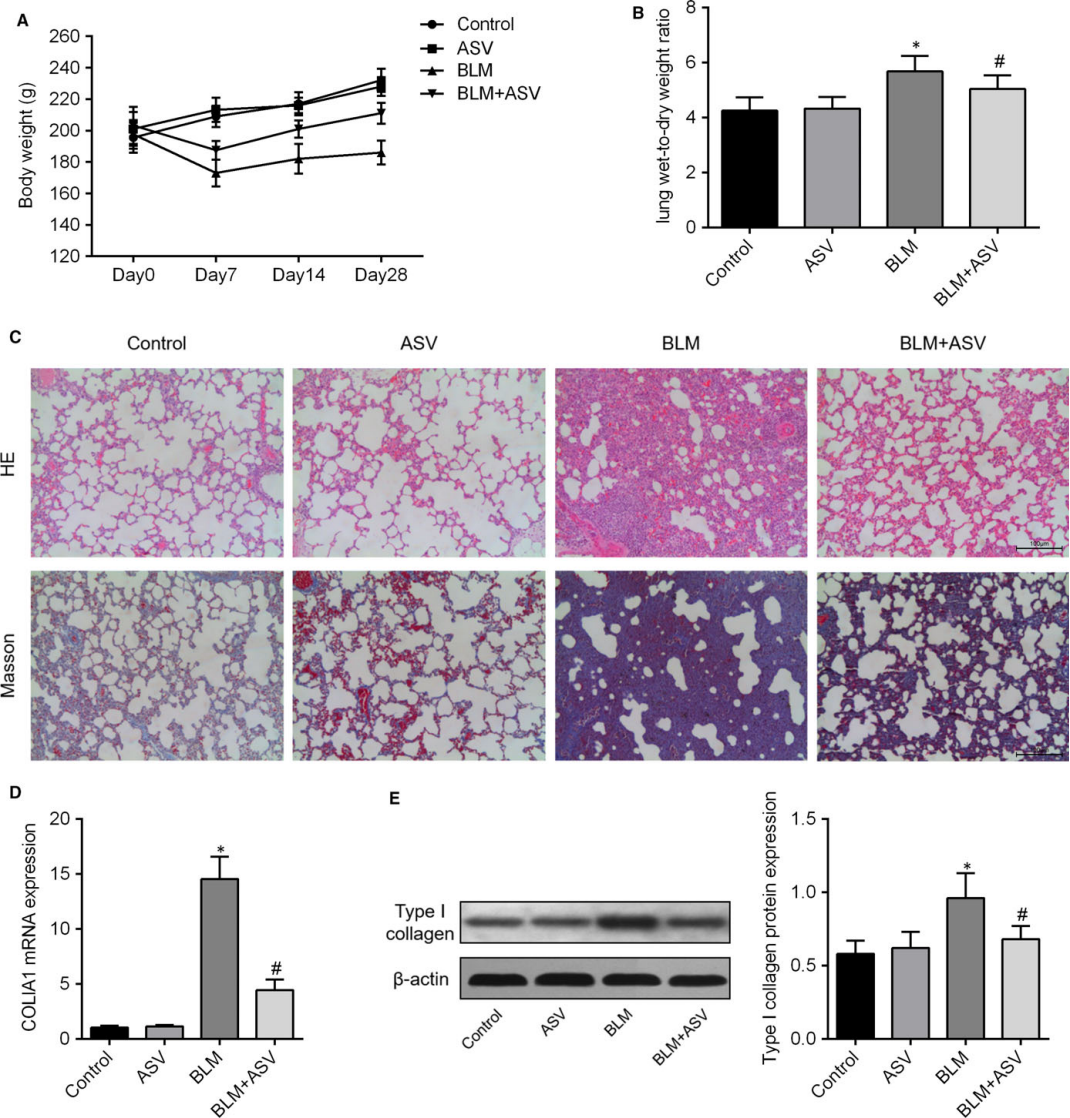
Astragaloside IV‐treated mice are protected against bleomycin‐induced pulmonary fibrosis. Body weight (A) and lung wet‐to‐dry‐weight ratio (B) of each rat subjected to saline or bleomycin at 30 mL/kg for 28 d with or without 20 mg/kg Astragaloside IV treatment (n = 5/group). Representative micrographs (100×) with hematoxylin and eosin staining and Masson's trichrome staining of paraffin lung sections (C). The expression level of COLIA1 mRNA detected by qPCR (n = 5/group). Representative Western blot of Type I collagen protein and densitometrically quantified data of Type I collagen/GAPDH expression ratio. *P < .05 vs control; ^#^ *P* < .05 vs BLM

Following intratracheal spray of BLM, a significant weight loss and increase in the lung wet‐to‐dry weight ratio were observed. But, BLM‐induced weight loss and the increase in the wet‐to‐dry weight ratio were reversed by ASV treatment (Figure 1A,B). H&E and Masson's trichrome staining showed pronounced diffuse fibrosis and lymphocyte infiltration and amounts of collagen were deposited in BLM group as compared with control group. Whereas, fibrosis showed apparent symptomatic relief in ASV treatment group compared with the BLM group (Figure 1C). In addition, compared with controls, the expression of collagen I mRNA and protein was also markedly increased caused by BLM. And 20 mg/mg ASV alleviated collagen deposition significantly (Figure 1D,E). These results suggest that ASV attenuated BLM‐induced pulmonary fibrosis.

### 3.2 ASV attenuated oxidative stress and inflammation in BLM‐induced pulmonary fibrosis

**Figure 2:**
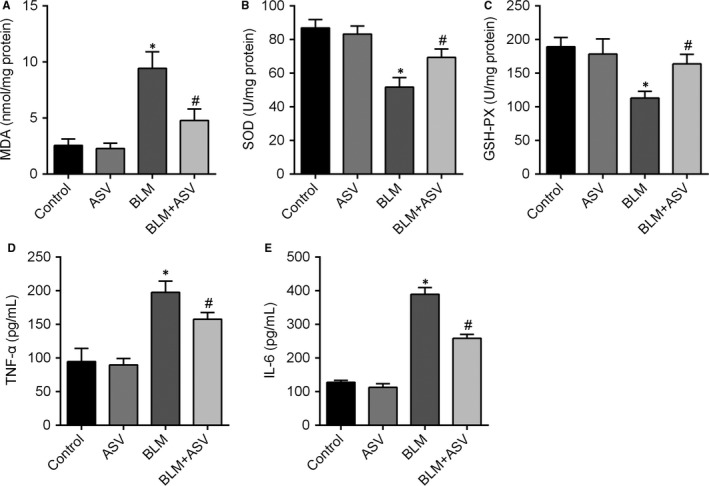
Inhibition of bleomycin‐induced oxidative stress and inflammation by the treatment with Astragaloside IV. Twenty‐eight days after bleomycin treatment, the markers of oxidative stress MDA (A), SOD (B), GSH‐PX (C), and the markers of inflammatory response TNF‐α (D) and IL‐6 (E) expression from the lungs of control group and those subjected to 30 mL/kg bleomycin with or without 20 mg/kg Astragaloside IV were measured. *P < .05 vs control; ^#^ *P* < .05 vs BLM

In order to validate the anti‐oxidative effects of ASV, oxidative stress was evaluated by detecting the levels of MDA, SOD, GSH‐PX and inflammatory responses by the levels of TNF‐α and IL‐6 in pulmonary tissue. In the BLM group, the levels of MDA were significantly elevated while SOD and GSH‐PX were reduced. In contrast, treatment with ASV dramatically decreased the levels of MDA and increased the levels of SOD and GSH‐PX (Figure 2A‐C). As for the inflammatory responses, BLM caused great increase in TNF‐α and IL‐6 levels, but the addition of ASV simultaneously suppressed the release of cytokines (Figure 2D,E). These results indicate the inhibitory effects of ASV on BLM‐induced oxidative stress and inflammation in pulmonary fibrosis.

### 3.3 ASV suppressed BLM‐induced EMT in pulmonary fibrosis in vivo

**Figure 3:**
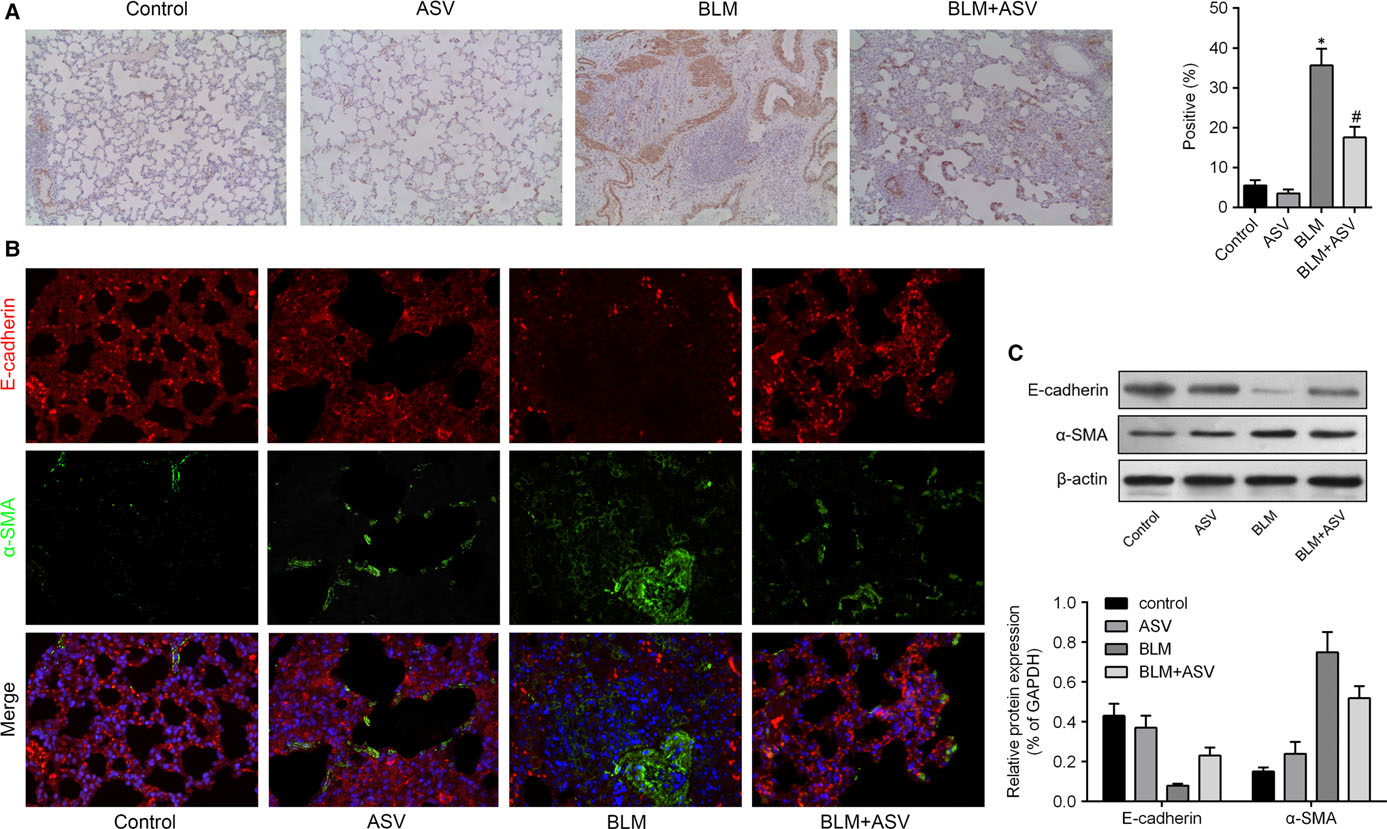
Astragaloside IV inhibited Bleomycin stimulated epithelial‐mesenchymal transition (EMT) in pulmonary fibrosis. A, Immunostaining of α‐SMA in lung tissue obtained from each group at 100 × magnification. B, Representative images of immunofluorescence double staining showing the overlap of E‐cadherin (red) and α‐SMA (green) accompanied with nuclei stained by DAPI (blue) in lung tissue sections. C, Expression of E‐cadherin and α‐SMA in the lung tissues as detected by Western blot analysis. *P < .05 vs control; ^#^ *P* < .05 vs BLM

To investigate the occurrence of EMT during lung fibrosis, the expression levels of α‐SMA and E‐cadherin were analysed using immunohistochemistry, immunofluorescence and western blot analyses. BLM treatment significantly increased the levels of α‐SMA in lung tissue (Figure 3A). Likewise, our immunofluorescence study further demonstrated that BLM treatment down‐regulated E‐cadherin expression and up‐regulated α‐SMA expression, when compared with control cells (Figure 3B). Western blot analysis of α‐SMA and E‐cadherin further validated the above results (Figure 3C). Above data suggested a decrease or variation in epithelial cells and an increase in myofibroblast cells during lung fibrosis. However, treatment with ASV reverses the progression of BLM‐induced EMT, decreased α‐SMA expression and increased E‐cadherin expression (Figure 3A‐C).

### 3.4 ASV inhibited TGF‐β1‐mediated hyperphosphorylation and inactivity of FOXO3a in pulmonary fibrosis

**Figure 4:**
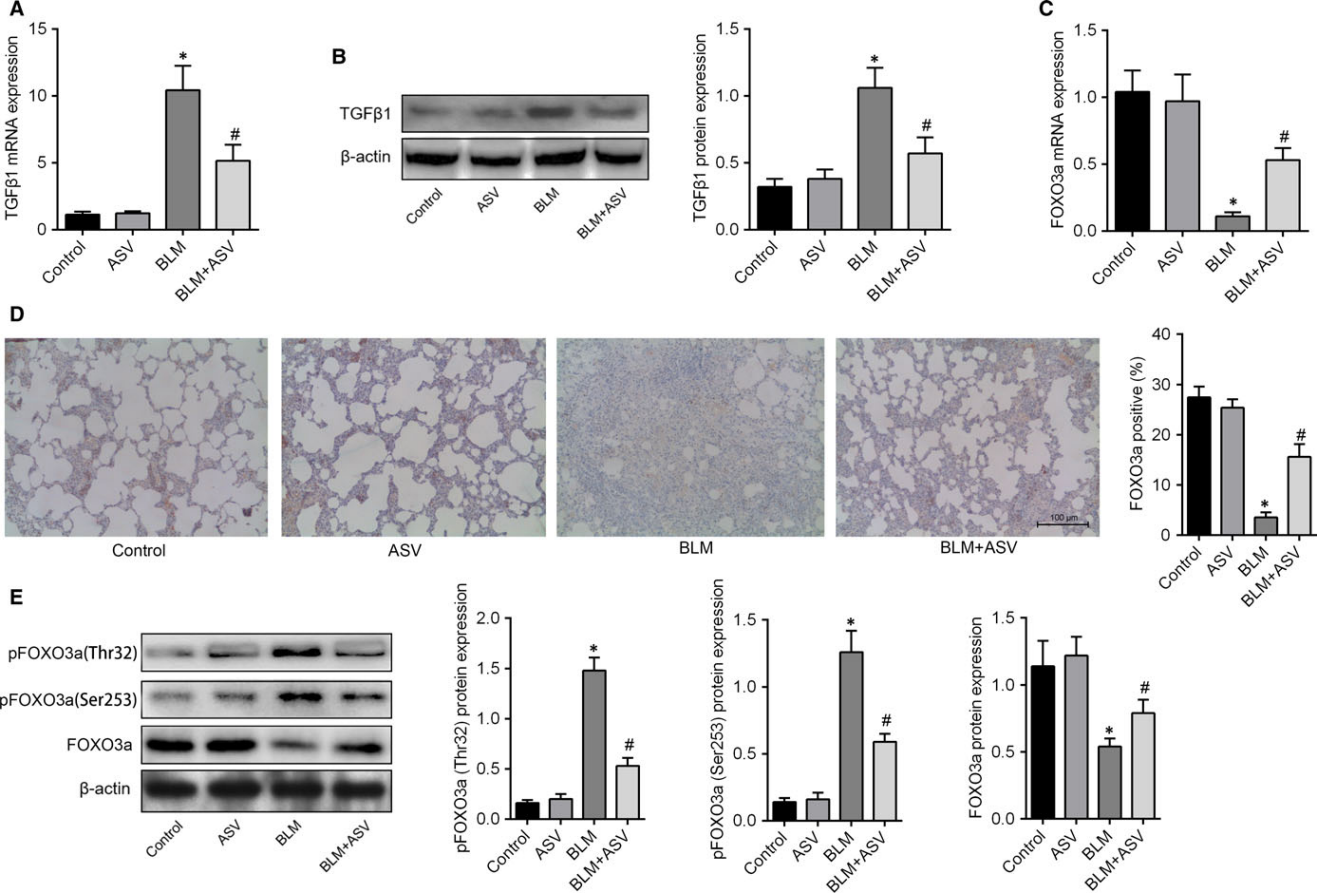
Astragaloside IV suppressed Bleomycin induced upregulation of TGF‐β1 and hyperphosphorylation and inactivity of FoxO3a. A, relative expression of TGF‐β1 mRNA in groups as measured by qPCR. B, Representative Western blots of TGF‐β1 and densitometry quantified data of TGF‐β1‐to‐β‐actin expression ratio. C, relative expression of FoxO3a mRNA in each group. D, Immunocytochemistry staining of FoxO3a in bleomycin or saline‐treated rats with or without ASV. E, Representative Western blots and densitometry quantified data of p‐FOXO3a (Thr32), p‐FOXO3a (Ser253) and FOXO3a protein levels. β‐actin was used as the internal control. *P < .05 vs control; ^#^ *P* < .05 vs BLM

TGF‐β1 plays a key role in the progression of pulmonary fibrosis. We thus investigated whether the inhibitory effects of ASV on pulmonary fibrosis was involved in its regulation of TGF‐β1 levels. BLM instillation significantly increased TGF‐β1 expression in lung tissue. As expected, we found that ASV treatment significantly alleviated BLM‐induced upregulation of TGF‐β1 both at mRNA and protein level (Figure 4A,B). We subsequently tested whether ASV was involved in the downstream target genes of TGF‐β1. FOXO3a was a critical downstream integrator of TGF‐β1 in human lung fibroblasts and intriguing, we identified that BLM notably down‐regulated the expression of FOXO3a mRNA (Figure 4C). Although ASV treatment didn't influence the transcription of FOXO3a, using immunohistochemistry, we found ASV significantly inhibited the decrease in FOXO3A protein caused by BLM (Figure 4D). Changes of FOXO3A activity were assessed by measuring the phosphorylation status of Thr32 and Ser253 of FOXO3a (Figure 4E). The levels of phosphorylated FOXO3a at Thr32 (Figure 4F) and Ser253 (Figure 4G) were significantly higher in BLM group as compared to the control and ASV group. However, co‐treatment with BLM + ASV significantly inhibited the hyperphosphorylation of FOXO3a thus to inhibit the inactivity of FOXO3a caused by BLM (Figure 4H),

### 3.5 ASV suppressed FOXO3a phosphorylation in TGF‐β1‐induced EMT via PI3K/Akt

**Figure 5:**
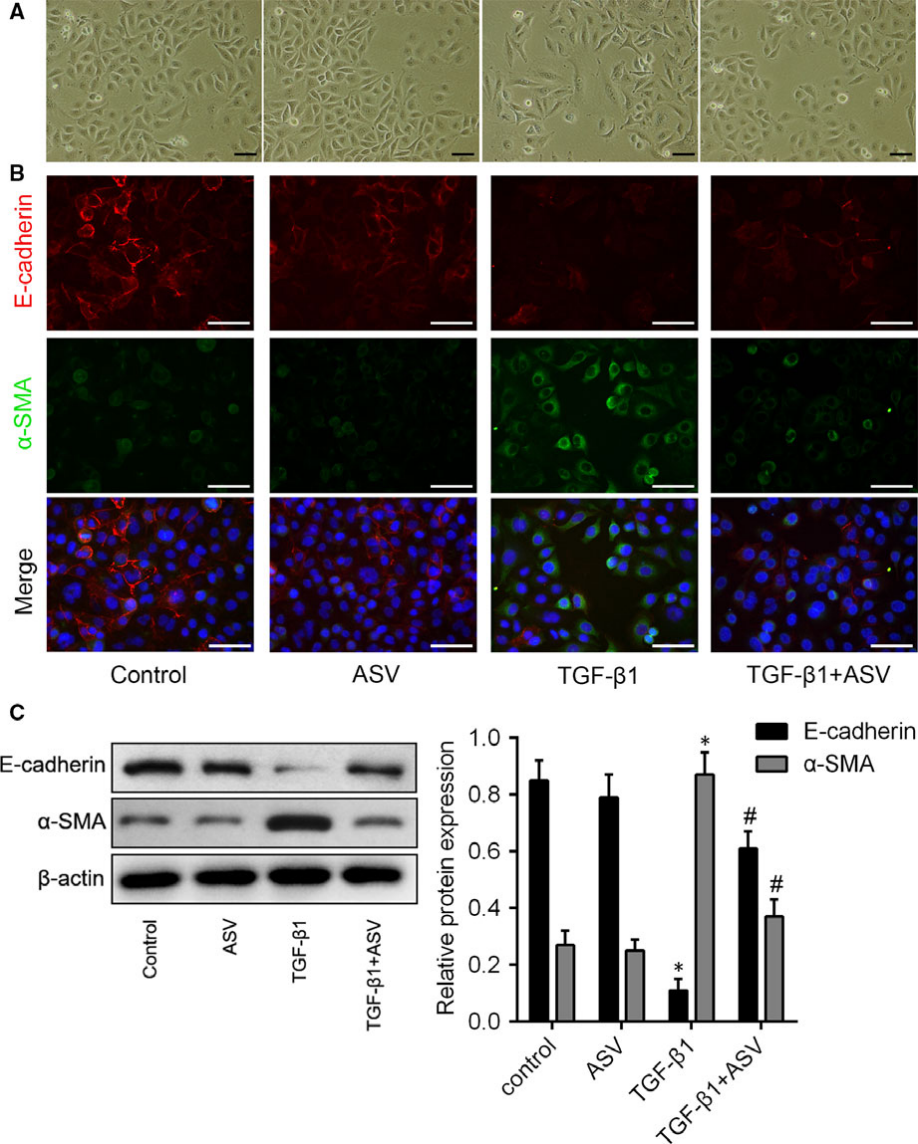
Astragaloside IV inhibited TGF‐β1 induced epithelial‐mesenchymal transition in pulmonary epithelial cells. A, TGF‐β1 treatment induces more elongated morphological shape and increases scattering, while addition of ASV suppressed these changes. B, Immunofluorescence double staining showing the overlap of E‐cadherin (red) and α‐SMA (green) in A549 cells in different groups. C, Western blots analysis of α‐SMA and E‐cadherin protein in each group. *P < .05 vs control; ^#^ *P* < .05 vs TGF‐β1

**Figure 6:**
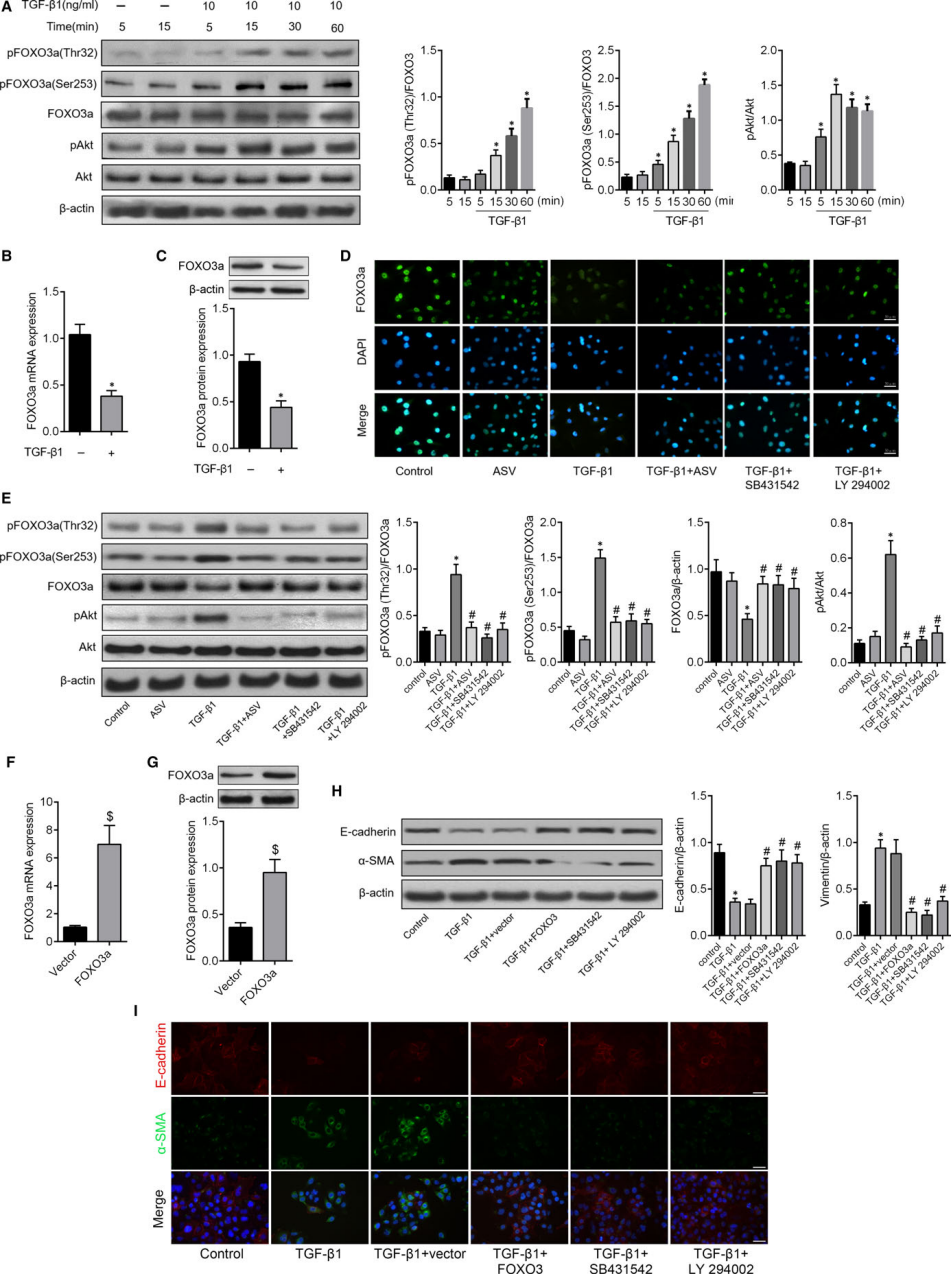
Astragaloside IV suppressed TGF‐β1/PI3K/Akt induced FOXO3a inactivity to reverse EMT in A549 cells. A, Western blots analysis of p‐FOXO3a (Thr32), p‐FOXO3a (Ser253), FOXO3a, p‐Akt and Akt protein levels. β‐actin was used as the internal control. B, Relative expression of FOXO3a mRNA level in A549. C, The expression levels of FOXO3a protein were tested by western blot analysis. D, Immunostaining of FOXO3a in A549 cells treated with 10 ng/mL TGF‐β1, 100 μg/mL ASV, 10 μmol/L SB431542 (a TGF‐β1 inhibitor) and or 20 μmol/L LY 294002 (a PI3K/Akt inhibitor). E, p‐FOXO3a (Thr32), p‐FOXO3a (Ser253), FOXO3a, p‐Akt and Akt expression via western blot following treatment with PBS, ASV, TGF‐β1, TGF‐β1 + ASV, TGF‐β1 + SB431542 or TGF‐β1 + LY 294002. F, Relative expression of FOXO3a mRNA in groups transfected with pcDNA‐3.1 + FOXO3a or pcDNA‐3.1 constructs. G, FOXO3a protein expression following transfection with a FOXO3a‐expressing plasmid. H, Western blots analysis of E‐cadherin and α‐SMA in indicated groups. I, Double‐labelled immunofluorescent staining was performed to examine the expression of E‐cadherin (red) and α‐SMA (green) in A549 cells in different groups. Scale bar = 50 μm *P < .05 vs control; ^#^ *P* < .05 vs TGF‐β1; ^$^ *P* < .05 vs Vector

It has been widely investigated that TGF‐β1 can induce and promote EMT. Similarly, we found that the TGF‐β1 treatment induced disappearance of intercellular junction and spindle‐like appearance were obviously reversed by ASV co‐treatment (Figure 5A). Using double‐labelled immunofluorescence assay, we further found that in A549 cells, TGF‐β1 increased the expression of α‐SMA, whereas the epithelial cell marker, E‐cadherin expression was decreased (Figure 5B). Addition of 100 μg/mL ASV significantly reversed above changes. The similar results can be found by western blot analysis in A549 cells (Figure 5C). To further uncover the potential mechanism of inhibitory effects of ASV on TGF‐β1‐stimulated EMT in pulmonary epithelial cells, we accessed the role of TGF‐β1 on the expression of Akt and FOXO3a. Stimulation with TGF‐β1 for 15 minutes significantly increased FOXO3a phosphorylation at the Thr32 site, whereas phosphorylation at the Ser253 site started after 1 hour. Along the same line, increased phosphorylation of Akt was also observed (Figure 6A). And interestingly, treatment with TGF‐β1 for 24 hours resulted in significant decreased FOXO3a expression both at mRNA (Figure 6B) and protein levels (Figure 6C). Immunofluorescence revealed that TGF‐β1 induced retention of FOXO3 in the cytoplasm, whereas ASV treatment reversed the inactivity of FOXO3a caused by TGF‐β1. Meanwhile, TGF‐β1 inhibitor SB431542, as well as PI3K/Akt inhibitor LY294002 reversed TGF‐β1‐induced decrease in FOXO3a (Figure 6D). We then identified that ASV strongly inhibited FOXO3a phosphorylation both at the Thr32 site Ser253 and site, accompanied with the restoration of FOXO3a and inhibition of phosphorylation of Akt. Inhibition of the upstream TGF‐β1/PI3K/Akt pathway using the corresponding inhibitors achieved the same results (Figure 6E). Furthermore, a gain of function analysis was performed to further validated TGF‐β1‐induced EMT through the phosphorylation of PI3K/Akt and subsequent inactivity of FOXO3a. As shown in Figure 6F,G, the expression levels of FOXO3a mRNA and protein were restored by transfected with a pcDNA3.1 + FOXO3a. Moreover, western blot and immunofluorescence assay showed that the expression of E‐cadherin was increased, whereas α‐SMA was decreased in TGF‐β1 + FOXO3a group as compared to that in the TGF‐β1 + vector group. The similar trends were observed after treatment with SB431542 or LY294002 (Figure 6H,I). These data indicated that ASV suppressed TGF‐β1/PI3K/Akt/FOXO3a‐mediated EMT in pulmonary epithelial cell.

## 4 DISCUSSION

In the present study, we further validated the protective effects of ASV on IPF. Our results, performed in a rat model of pulmonary fibrosis and cultured human type II alveolar epithelial cells, provided assured evidence of the important role of TGF‐β1‐mediated FOXO3a in controlling EMT from epithelial cells to myofibroblasts. The results that ASV activated FOXO3a in TGF‐β1‐induced EMT via inhibition of PI3K/Akt pathway mechanically explained the inhibitory role of ASV in pulmonary fibrosis fibrogenesis and indicated that ASV could be served as a potential therapeutic strategy for IPF.

The main histopathological features of IPF is aggregates of proliferating fibroblasts and myofibroblasts—fibroblastic foci—within a myxoid‐appearing matrix, resulting in scarring and destruction of the lung architecture. And this fibrotic response is driven by abnormally activated alveolar epithelial cells.^23^ The activated myofibroblasts, always considered transformed from epithelial cells and displayed a different phenotype as compared to normal lung fibroblasts, produce extracellular matrix proteins which contribute to the progression of IPF.^24^ As expected, destroyed alveolar epithelial cells and a great accumulation of ECM were observed in BLM stimulated lungs when compared to the control and ASV‐treated groups. In contrast, when treated with ASV, a natural saponin with a potential anti‐fibrotic property, the above pathological changes were significantly attenuated. This was consistent with a previous study describing that ASV ameliorated BLM‐induced ECM deposition and decreased the levels of hydroxyproline and type III collagen.^17^ We also showed ASV decreased the level of type I collagen. Inflammation and oxidative stress also play a pivotal role in pulmonary fibrosis.^25^ Studies supported that anti‐inflammation or antioxidant treatment strategies may achieved improvement in pulmonary fibrosis.^26^ We found the levels of inflammatory cytokine (TNF‐α and IL‐6) and markers of oxidative stress (MDA, SOD and GSH‐PX) in BLM‐treated rats were dramatically increased detected, whereas ASV prevented BLM‐induced elevation of inflammatory cytokine and oxidative stress. These observations were in attendance with the Yu's results.^16^

TGF‐β1 is an attractant for fibroblasts and monocytes/macrophages and stimulates these cells to synthesize the pro‐inflammatory and fibrogenic cytokines. It is also an inducer of ECM production.^27^ TGF‐β1 was elevated after bleomycin treatment, as was consistent with previous investigations. TGF‐β1 plays an important role in pulmonary fibrosis in that it mediates the differentiation of fibroblast.^28^ The best‐studied and central player in EMT is TGF‐β1. As a result of the notion that EMT directly contributes to myofibroblast accumulation in the lung,^5^, ^29^ groups of studies tried to find efficient approaches to inhibit or reverse this progress mediated by TGF‐β1.^30^, ^31^, ^32^ We for the first time identified the increased TGF‐β1 production was potently suppressed by ASV in BLM‐induced IPF. Based on this finding, we further tried to reveal the role of ASV on the downstream targets of TGF‐β1. In fact, activation of several non‐Smad signalling pathways by TGFβ1, including mitogen‐activated protein kinase (MAPK), AKT, and non‐Smad substrates for the TGFβ1 receptor, generally promotes aspects of EMT.^33^, ^34^, ^35^ A series of studies identified TGF‐β1 initiates activation of PI3K/Akt signaling and phosphorylation of Akt was recognized to inhibit the function of FOXO transcription factors, especially FOXO3.^36^, ^37^, ^38^ Consistently, our results revealed that Akt phosphorylates and inactivates FOXO3a, leading to the retention of FOXO3a in the cytoplasm and inhibition of target gene transcription.^39^

FOXO3a alteration is closely linked to pulmonary fibrosis. FOXO3 is hyperphosphorylated and down‐regulated in IPF fibroblasts. Moreover, knockdown of FOXO3 in normal fibroblasts reproduces IPF fibroblast phenotype.^21^ In the current study, we found ASV or inhibition of PI3K/Akt pathway could significant suppress TGF‐β1 caused FOXO3 hyperphosphorylation. Thus, we infer that ASV could inhibit TGF‐β1‐induced activity of PI3K/Akt to reverse the inactivity of FOXO3, therefore to suppress EMT. Our hypothesis was also supported by a previous study showing ASV could up‐regulate Smad7 in the TGF‐β1/Smad signaling pathway to inhibit the progress of EMT in peritoneal mesothelial cells.^13^ Importantly, Li et al^17^ identified that ASV inhibited the increased α‐SMA in the BLM‐induced fibrosis. Our work found that overexpression of FOXO3a in vitro had the similar effects with TGF‐β1 or PI3K/Akt inhibitor to reverse TGF‐β1‐induced EMT in epithelial cells. Taken together, the results suggested that FOXO3a plays an important role in the protective effects of ASV on IPF.

**Figure 7:**
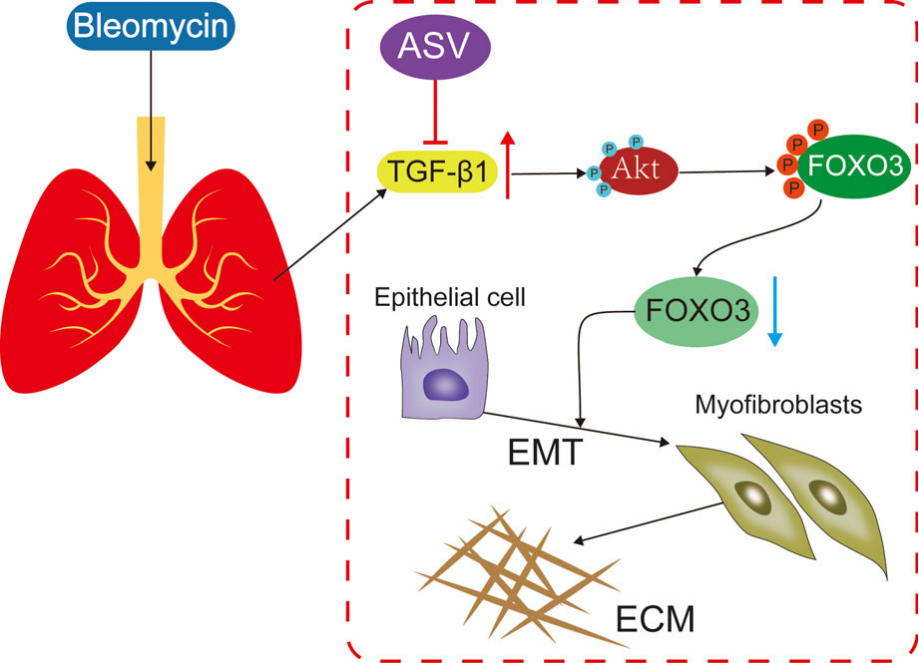
Astragaloside IV protected against bleomycin‐induced pulmonary fibrosis through suppressing TGF‐β1‐mediated EMT. Astragaloside IV inhibited TGF‐β1 induced activity of PI3K/Akt pathway independent inactivity of FOXO3a in pulmonary epithelial cell

In conclusion, our integrated approach demonstrates that ASV treatment suppressed TGF‐β1/PI3K/Akt pathway to active FOXO3a, thus to prevents EMT in BLM‐induced pulmonary fibrosis (Figure 7).

## CONFLICTS OF INTEREST

The authors declare that they have no conflicts of interest.
